# The cardiovascular complications in COVID-19: Focus on acute cardiac injury

**DOI:** 10.12669/pjms.37.3.4063

**Published:** 2021

**Authors:** Sahrai Saeed, Ronak Rajani

**Affiliations:** 1 Sahrai Saeed, Department of Heart Disease, Haukeland University Hospital, Bergen, Norway; 2 Ronak Rajani Cardiothoracic Centre, Guy’s & St Thomas’ NHS Foundation Trust, London, United Kingdom. School of Biomedical Engineering and Imaging Sciences, King’s College London, United Kingdom

**Keywords:** Acute cardiac injury, Antihypertensive treatment, Cardiovascular complications, Coronavirus disease 2019, COVID-19, Hypertension, Prognosis

## Abstract

At the end of 2019 a novel coronavirus was identified in Wuhan, China. The disease caused by the severe acute respiratory distress syndrome coronavirus 2 (SARS-CoV-2) was designated COVID-19 (corona virus disease 2019) by the World Health Organization in early 2020. Up to 80% of patients with COVID-19 experience mild symptoms with severe or critical disease occurring in the remaining 20%. Severe disease is manifested by the development of pneumonia, hypoxia and radiographic lung involvement while critical disease indicates multiorgan involvement with significant respiratory or cardiac compromise. The current estimated case fatality rate from COVID-19 is approximately 1%. Epidemiological studies have shown that advanced age, male gender, previous chronic lung disease, cardiovascular and kidney disease, obesity and diabetes are risk factors for the severity of disease course. In the current focused review, we present an overview of the acute cardiovascular complications of COVID-19, their detection and impact upon prognosis.

## INTRODUCTION

The first confirmed case of coronavirus disease 2019 (COVID-19) was registered in Wuhan, China one year ago. Soon after its outbreak, the disease rapidly spread to the entire world and was declared as pandemic by World Health Organization on 11^th^ March 2020. As of the 20^th^ December 2020, there have been 76,718,430 confirmed cases with 1,639,942 deaths worldwide.^1^ Routine hematological, biochemical and serological tests, as well as radiographic examinations are essential to confirm the diagnosis and assess progression in SARS-CoV-2 infected patients.^2^ Among these, a positive PCR for SARS-CoV-2 infection confirms the diagnosis. Currently, there are no proven curative treatments to the disease with supportive therapy being the mainstay of treatment. The recent regulatory approval of the Pfizer, AstraZeneca and Moderna COVID-19 vaccines and their introduction to the United Kingdom and US offer some hope that the trajectory of the pandemic may be altered over the next 12 months as further countries adopt vaccination programmes.

COVID-19 infection is associated with a number cardiovascular (CV) comorbidities that include hypertension, obesity, diabetes mellitus, coronary artery disease and congestive cardiac failure. Although it remains uncertain whether these observations are simply related to age, it is evident that patients who develop severe disease are more vulnerable in the presence of these comorbidities. Reported case fatality rates vary between countries and this likely reflects methods of reporting. Despite this, men, elderly and ethnic minorities appear to be disproportionately affected by COVID-19 with a more severe disease course and case fatality rate. Up to 16% of patients develop a severe illness during hospitalization with 5% of these patients requiring admission to Intensive care and 2.3% requiring intubation. The case fatality of COVID-19 infection is currently estimated to be 1%.^3^

The clinical manifestations of mild COVID-19 include fever, dry cough and myalgia. Pneumonia is the most serious consequence of COVID-19 and is often accompanied by additional symptoms of cough, chest pain, breathlessness and fatigue. This is confirmed by a chest X-ray showing bilateral diffuse interstitial changes and ground glass opacification and/or consolidation on computed tomography. With multiorgan involvement, elevated liver enzymes, renal dysfunction, disseminated intravascular coagulation, lymphopenia, thrombocytopenia, hypercoagulability, neurological complications and cardiac involvement can ensue.

Studies from Italy and the US have shown that more than 40% of COVID-19 patients presented with any CV disease.^4^-^5^ Among these hypertension, diabetes, hyperlipidemia, coronary artery disease, heart failure and atrial fibrillation were common comorbidities.^4^ COVID-19 patients with pre-existing CV disease have higher mortality rates, thromboembolic events, septic shock rates, and need for ICU treatment with a ventilator support compared with those who do not have a history of cardiac disease.^4^-^5^

### Risk factors in COVID-19:

Male gender, higher age, chronic lung disease, kidney disease, systemic hypertension, diabetes mellitus, obesity and metabolic syndrome have been identified as risk factors for COVID-19 severity. There may be a bidirectional association between hypertension and COVID-19. A large body of publications over the past six months have indicated that hypertension is independently associated with severity of COVID-19.^5^-^6^ The fact that coronavirus in human bodies binds to epithelial lung cells through the peptide angiotensin-converting enzyme (ACE) 2, led to safety concerns of antihypertensive treatment with RAAS blockers (renin-angiotensin-converting enzyme inhibitors [ACE] and angiotensin receptor blockers [ARBs] in COVID-19 patients. These concerns are now to some extent diminished with the emergence of studies showing that anti-hypertensive treatment with RAAS blockers may not worsen the severity of COVID-19.^7^-^9^ With this reassurance, hypertension in COVID-19 patients can be treated according to international guidelines for management of hypertension in adults.

### Acute CV complications in COVID-19:

**Fig.1:**
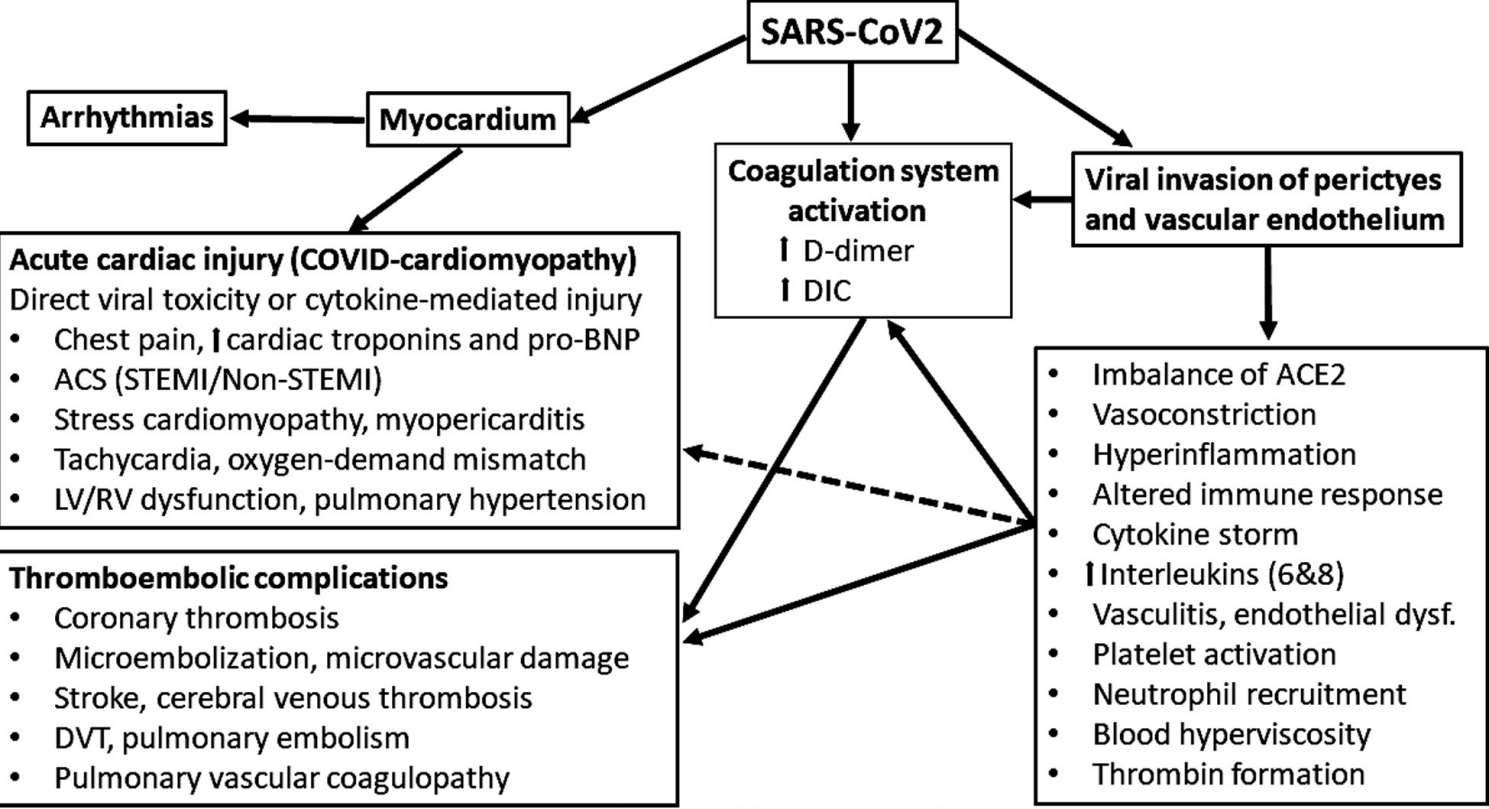
An overview on cardiovascular complications in COVID-19 from a clinical point of view.

Acute CV complications in active COVID-19 disease include chest pain, elevated cardiac biomarkers, tachycardia, cardiac injury (acute coronary syndromes, stress cardiomyopathy, myopericarditis/myocarditis), left ventricular (LV) and right ventricular (RV) failure, pulmonary hypertension, thromboembolic events, arrhythmias, hemodynamic instability and sudden death (Table-I, Fig.1)^10^-^12^ Studies show that cardiac involvement in COVID-19 is highly prevalent with a broad spectrum of clinical manifestations. These range from elevated myocardial enzyme levels in 54%, cardiac dysfunction in 41% and acute cardiac injury in 9% patients.^13^ Acute cardiac injury in COVID-19 patients is associated with poor prognosis. The etiology may be different from the conventional acute coronary obstructions/plaque ruptures with subsequent STEMI and Non-STEMI.^14^ SARS-CoV-2 can damage the myocardium (both RV and LV) and coronary circulation directly as a result of its toxic effects, or indirectly by increasing the propensity to thrombus formation and acute coronary artery obstruction (STEMI or Non-STEMI).^15^ Other forms of acute cardiac injury in these patients include stress cardiomyopathy, atypical myocardial infarction due to myocardial demand-supply mismatch secondary to tachycardia/stress, microvascular embolization, endothelial dysfunction and cytokine storms by an altered immune- and inflammatory response.^11^, ^15^ An acute myopericarditis may also be accompanied by cardiac tamponade requiring urgent pericardial drainage.^16^

**Table I:** The cardiovascular complications in COVID-19.

***Acute CV complications***
***Acute cardiac injury***
Elevated biomarkers (troponins and pro-BNP)
Acute cardiac injury
Chest pain
Tachycardia
Acute coronary syndromes (STEMI/Non-STEMI
Stress cardiomyopathy (Takotsubo syndrome)
Pericardial effusion/Tamponade
Pericarditis, myopericarditis, myocarditis
LV dysfunction
Cardiogenic shock
RV dysfunction
Elevated systolic pulmonary artery pressure/PAH
Sudden cardiac death
***1.Thromboembolic complications***
Intracardiac thrombus
Pulmonary embolism
Deep venous thrombosis
Stroke
***2.Arrhythmias/conduction abnormalities***
Atrial fibrillation, ventricular arrhythmias, heart block
Post-COVID
***1.Exaggerated CV response***
Persistently elevated blood pressure
Higher resting heart rate/sinus tachycardia
***2.Persistent myocardial inflammation***

### Diagnostic approach:

A targeted cardiac evaluation is recommended in patients with suspected cardiac involvement suggested by heart failure symptoms, cardiac arrhythmias or otherwise unexplained electrocardiographic (ECG) findings. This should take the form of a physical examination, serum Troponin levels and serum brain natriuretic peptide (BNP) measurement in patients with suspected heart failure. Serum Troponins have been found to be elevated in up to 36% of patients with COVID-19 with higher values being associated with a worse clinical course.^17^-^18^ Patients with a mild rise in Troponin that subsequently declines over a few days is generally associated with a favorable prognosis compared with those patients with a moderate Troponin level which subsequently rises with elevations in other biomarkers (Interleukin 6, ferritin, lactate dehydrogenase and D-dimer).^19^-^20^ BNP levels are commonly elevated in COVID-19 patients (12.9%)^21^ and carries an increased risk of mortality with a HR of 5.11 (95% CI 3.50-7.47). ECG findings are variable and include atrial tachyarrythmias, right bundle branch block (RBBB) and repolarization abnormalities in up to 29% of individuals.^22^ The ECG features associated with a poorer prognosis include RBBB, interventricular conduction delays, atrial premature beats and nonspecific repolarization abnormalities.

To assess acute cardiac injury or CV complications in COVID-19 patients, a bedside transthoracic echocardiogram is recommended both by the European and American Societies of Cardiology.^23^-^24^ In critically ill COVID-19 patients, tricuspid annular plane systolic excursion (TAPSE), may be preserved until the late stage, while radial function of the RV may be reduced. 25-26 Furthermore, other markers of systolic RV function such as fractional area change divided by RV systolic pressure and RV longitudinal strain have been identified as important markers of disease severity and strong predictors of mortality in COVID-19.^27^ Overall, cardiac abnormalities may be observed in 50% of all patients undergoing echocardiography.^28^

The presence of serum biomarkers of myocardial injury and echocardiographic abnormalities indicates a subset of patients with higher mortality than those with elevations in BNP or Troponins alone.^29^

### Thromboembolic complications in COVID-19:

COVID-19 is a hypercoagulable state and result in thromboembolic complications both in venous and arterial system. Common thromboembolic complications in COVID-19 are intracardiac thrombus, stroke, deep vein thrombosis and pulmonary embolism (Table-I, Fig.1) 30-32

### Post-COVID-19 CV response:

After recovery from COVID-19 disease, some patients may develop sustained sinus tachycardia and elevated BP. Although the exact cause of this phenomenon is unknown, it has been speculated that prolonged periods of mechanical ventilation, the use of inotropic drugs, fluid overload, increased adrenergic tone, inflammation (interleukins) and hyper-reninemia^33^-^34^ may have some accountability. Furthermore, Cytokine storms, similar to their acute impact on cardiac injury, may also be responsible for short- and long term CV consequences such as persistently elevated BP and high resting heart rate.

Finally, reports also indicate that COVID-19 is associated with diabetic complications (manifested as new onset diabetes with diabetic ketoacidosis or poor diabetic control) and acute kidney injury, which have been described in detail elsewhere.^35^

### Future directions:

During the ongoing pandemic, the main emphasis has been the diagnosis and treatment of acute cardiac injury. The true burden of CV complications and their long-term consequences is likely only to emerge once longer-term data is available on survivors of COVID-19 who had confirmed cardiac involvement. The results of current and future studies will be required to fully understand a number of outstanding questions relating to cardiac involvement in patients with COVID-19:

1.The extent of RV dysfunction and its relationship with elevated cardiac biomarkers.
2.The reversibility of LV and RV dysfunction, and pulmonary hypertension.
3.The prevalence of arrhythmias varied from in different studies from 17%^22^ to 29% (36). Hence, the prevalence, types and the exact mechanisms of arrhythmias and conduction abnormalities need to be explored in larger COVID-19 studies.
4.Assessment of subclinical LV dysfunction by speckle tracking echocardiography, and coronary flow reserve by advanced multimodality imaging.
5.The burden of COVID-endocarditis and subclinical valvular thrombosis.
6.What is the optimal clinical follow-up strategy for COVID-19 survivors with proven cardiac involvement? What should be the frequency of further transthoracic echocardiograms, and should clinical evaluation be accompanied by an assessment of cardiopulmonary function test with peak oxygen uptake, clinic and ambulatory blood pressure measurements.
7.Cardiac MRI has shown a high prevalence of myocardial tissue changes in patients who recovered from COVID-19.^37^ The impact of these changes on prognosis will need to be evaluated.
8.The prognostic value and precise mechanism of an exaggerated CV response in COVID-19 survivors.
9.Although some useful exercise prescriptions for general population during the COVID-19 pandemic have been described in the literature^38^ a well-structure cardiac rehabilitation program for COVID-survivors per se should be established.

## CONCLUSIONS

Coronavirus affects CV system either by its direct toxic effect or through cytokine storms mediated by altered inflammatory or immunological response. Acute cardiac injury, thromboembolic complications and arrhythmias are common in COVID-19 patients and portend worse prognosis. The long-term outcome of CV involvement in COVID-19 is not yet fully understood and should be evaluated in larger prospective studies in the future.

### Authors’ Contributions:

**SS and RR** wrote the manuscript and s approved the final submission.
